# MCT4 as a potential therapeutic target for metastatic gastric cancer with peritoneal carcinomatosis

**DOI:** 10.18632/oncotarget.9523

**Published:** 2016-05-20

**Authors:** Ji Yun Lee, InKyoung Lee, Won Jin Chang, Su Min Ahn, Sung Hee Lim, Hae Su Kim, Kwai Han Yoo, Ki Sun Jung, Haa-Na Song, Jin Hyun Cho, Sun Young Kim, Kyoung-Mee Kim, Soojin Lee, Seung Tae Kim, Se Hoon Park, Jeeyun Lee, Joon Oh Park, Young Suk Park, Ho Yeong Lim, Won Ki Kang

**Affiliations:** ^1^ Division of Hematology-Oncology, Department of Medicine, Samsung Medical Center, Sungkyunkwan University School of Medicine, Seoul, Korea; ^2^ Biological Research Institute, Samsung Medical Center, Sungkyunkwan University School of Medicine, Seoul, Korea; ^3^ Division of Hematology-Oncology, Department of Medicine, Korea University College of Medicine, Seoul, Korea; ^4^ Innovative Cancer Medicine Institute, Samsung Cancer Center, Samsung Medical Center, Seoul, Korea; ^5^ Department of Pathology and Translational Genomics, Samsung Medical Center, Sungkyunkwan University School of Medicine, Seoul, Korea

**Keywords:** gastric cancer, monocarboxylate transporter, glycolysis, prognosis

## Abstract

Monocarboxylate transporters (MCTs) play a major role in up-regulation of glycolysis and adaptation to acidosis. However, the role of MCTs in gastric cancer (GC) is not fully understood. We investigated the potential utilization of a new cancer therapy for GC. We characterized the expression patterns of the MCT isoforms 1, 2, and 4 and investigated the role of MCT in GC through *in vitro* and *in vivo* tests using siRNA targeting MCTs. In GC cell lines, MCT1, 2, and 4 were up-regulated with different expression levels; MCT1 and MCT4 were more widely expressed in GC cell lines compared with MCT2. Inhibition of MCTs by siRNA or AR-C155858 reduced cell viability and lactate uptake in GC cell lines. The effect of inhibition of MCTs on tumor growth was also confirmed in xenograft models. Furthermore, MCT inhibition in GC cells increased the sensitivity of cells to radiotherapy or chemotherapy. Compared with normal gastric tissue, no significant alterations of expression levels in tumors were identified for MCT1 and MCT2, whereas a significant increase in MCT4 expression was observed. Most importantly, MCT4 was highly overexpressed in malignant cells of acsites and its silencing resulted in reduced tumor cell proliferation and lactate uptake in malignant ascites. Our study suggests that MCT4 is a clinically relevant target in GC with peritoneal carcinomatosis.

## INTRODUCTION

Gastric cancer (GC) is the fourth most common cancer and the second leading cause of cancer deaths globally [1]. In advanced and metastatic gastric cancer, conventional chemotherapy with limited efficacy shows a median overall survival (OS) of less than 1 year [2, 3]. Despite recent advances in the genetic landscape of GC that have led to investigations into the application of targeted therapies, this progress has yet to translate into improved survival outcomes for GC [4, 5].

In rapidly growing cancer cells, oncogenes and hypoxia stimulate the glycolytic metabolism, which generates increased amounts of lactic and carbonic acids [6–8]. To avoid intracellular acidification and death, the intracellular pH is regulated as the influx and efflux of lactate are controlled by monocarboxylate transporter (MCT) [9]. The MCT family comprises 14 members, among which only the first 4 (MCT1, 2, 3, and 4) catalyze the proton-linked transport of metabolically important monocarboxylates, such as lactate, pyruvate, and ketone bodies [10]. Depending on the tissue and the species, MCT1 or MCT2 takes up lactic acid and ketone bodies for oxidation or lactic acid for gluconeogenesis [11]. MCT3 is uniquely expressed in the retinal pigment epithelium [12], whereas MCT4 is primarily expressed in highly glycolytic cells, where it is used to facilitate lactic acid effuse from the tissue [13].

It was recently shown that high expression of MCTs correlates with invasiveness and poor prognosis in several solid tumors including colorectal cancer [14], cervical cancer [15], breast cancer [16], and glioblastoma [17]. This discovery has spurred the development of MCT inhibitors with potential clinical applications. Mathupala et al. showed that MCT1 RNA interference causes cell death in glioma cell lines [18] and the specific MCT1/2 inhibitor AR-C155858 impaired proliferation of RAS-transformed fibroblasts *in vitro* and *in vivo* [19]. Furthermore, Radoslaw et al. revealed that AZD3965, as an MCT1 inhibitor, reduced tumor growth and increased intratumor lactate [20].

Considering the aggressiveness of GC, up-regulation of glycolysis and adaptation to acidosis might be key mechanisms of GC progression, such as cancer cell invasion and metastasis. However, the patterns of MCT expression in GC and the role of these transporters are still poorly understood [21]. In this study, we aimed to investigate the biological role of MCT1, 2, and 4 in advanced GC and to assess the potential of a targeted anti-cancer agent in GC.

## RESULTS

### MCTs are expressed in gastric cancer cell lines with distinct levels

**Figure 1:**
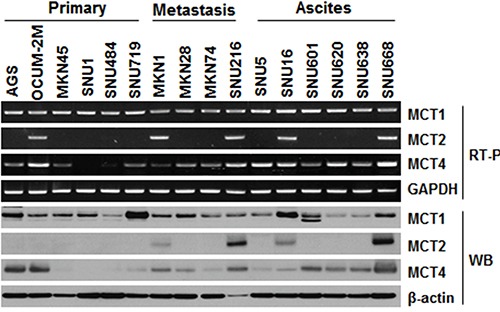
Expression levels of MCTs (MCT1, MCT2, and MCT4) in gastric cancer cell lines RT-PCR and western blot analysis were used to assess MCT expression in 16 gastric cell lines (primary, N = 6; metastasis, N = 4; ascites, N = 6). mRNA levels were normalized to GAPDH and protein levels to β-actin.

MCT protein expression was evaluated in 16 GC cell lines by reverse transcription polymerase chain reaction (RT-PCR) and western blot (Figure 1). Overall, there were different expression levels of MCT1, MCT2, and MCT4 in GC cell lines, though with different expression patterns. MCT1 was expressed in almost all GC cell lines, whereas MCT4 was primarily expressed in cell lines derived from metastasis or ascites. Expression of the MCT2 isoform was less common in GC cell lines than was expression of MCT1 and MCT4. Consistent with the observations in RT-PCR, the similar isoform-specific expression phenomenon was also observed by western blot assay.

### MCT knockdown affects cellular proliferation and lactate uptake

**Figure 2:**
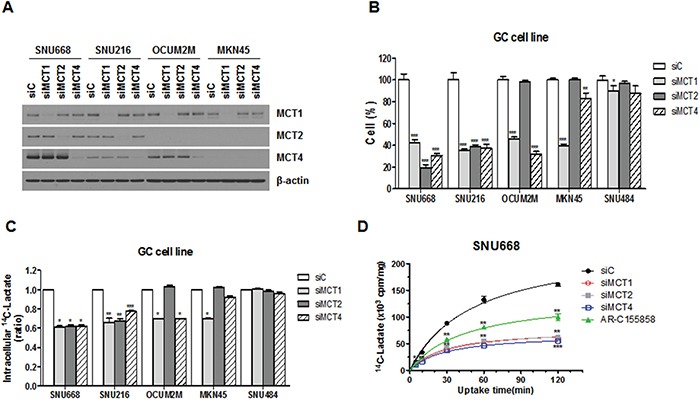
Effect of MCT inhibition on cell proliferation and lactate uptake **A.** Expression of MCTs in GC cell lines after transfection with siRNAs was analyzed by western blot analysis using antibodies to the proteins indicated. **B.** Cell proliferation was measured 72 hours after transfection with MCT siRNA (siMCT1, siMCT2, or siMCT4) or a negative control sequence (siC). The percentage of viable cells is shown relative to that of the untreated control. **C.** [^14^C]-L-lactate uptake was measured after knockdown of MCTs in GC cell lines. **D.** The effect of MCT inhibition on lactate uptake in the SNU668 cell line was evaluated over time and siC was used as a reference. Values represent the mean of 3 independent experiments. Data represent mean and standard deviation. *P < 0.05; **P < 0.01; ***P < 0.001.

We determined the ability of MCT down-regulation to inhibit cellular proliferation and lactate uptake (Figure 2). Western blot results confirmed that silencing of MCTs selectively decreased the MCTs expression in GC cell lines (Figure 2A). As MCTs were knocked down by siMCT1, 2, or 4 after 72 h, cell proliferation was significantly decreased in SNU668 and SNU216 cell lines, which highly expressed MCT1, 2, and 4 as compared with expression levels seen in the control group (Figure 2B). Growth of OCUM-2M cells was significantly inhibited by the presence of either siMCT1 or siMCT4, relative to the clones treated with the control siRNA, confirming the specific ablation of MCT1 and MCT4 (Figure 2B). When we transfected the MCT-negative SNU484 cells with the siMCTs, no significant growth alteration was observed (Figure 2B). Furthermore, a selective growth inhibition by siMCTs was observed for the MCT-positive breast cancer cell line (Supplementary Figure S1).

We next examined whether MCT siRNA functionally blocked lactate entry into cancer cells (Figure 2C and 2D). The intracellular lactate level was significantly lower in SNU668 cells treated with siMCT1, 2, or 4 (Figure 2C). In the case of MKN45 cells, only transfection with siMCT1 resulted in significantly decreased intracellular lactate levels, confirming the specific ablation of MCT1 (Figure 2C). To investigate the time-dependence of the uptake, SNU668 cells were incubated at room temperature for 5, 30, 60, and 120 min (Figure 2D). Overall, lactate uptake decreased over time with a significant decrease in cell lines treated with siMCTs or AR-C155858 (Figure 2D).

### MCT inhibitor decreases *in vivo* tumorigenesis

**Figure 3:**
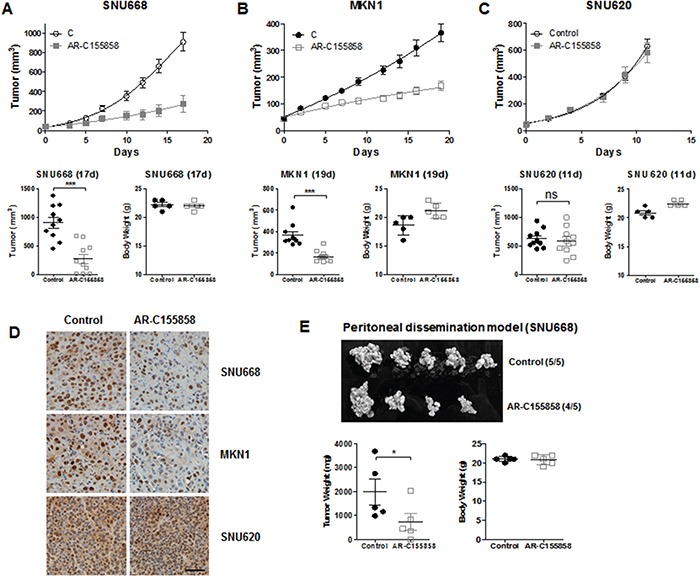
*In vivo* efficacy of MCT inhibition BALB/c nude mice were injected subcutaneously with GC cell lines. One week after injection, mice were treated 3 times per week with an intraperitoneal injection of 1 mg/kg AR-C155858. Upper panels show the time course of the growth and lower panels represent the mean tumor volume and standard deviation of SNU668 **A.** MKN1 **B.** and SNU620 **C.** tumors following administration of AR-C155858 (*vs.* PBS as a control). **D.** Proliferation in each type of xenograft (SNU668, MKN1, and SNU620) was analyzed by IHC with anti-PCNA antibody (Santa Cruz Biotechnology, PC-10, 1:2000). The brown staining in the nucleus is the PCNA signal. **E.** For the peritoneal dissemination model, mice were inoculated intraperitoneally with SNU668 cells. The upper panel shows the representative xenograft tumors resected on day 21, showing the difference in tumor volumes. Lower panels represent mean tumor volume and standard deviation. This experiment was repeated three times with similar results. *P < 0.05, ** *P* < 0.01, *** *P* < 0.001.

To further determine whether the MCT inhibition was relevant *in vivo*, we next treated mice bearing tumors derived from GC cells (Figure 3). We selected SNU668 and MKN1 as MCT1, 2, and 4-positive GC cell lines, and SNU620 as a MCT4-positive GC cell line. As an MCT inhibitor, we used the recently developed AR-C155858, which is specific to MCT1/2 [22, 23]. For this experiment, one week after subcutaneous inoculation with GC cell lines, mice were treated 3 times per week with an intraperitoneal injection of AR-C155858.

Treatment with AR-C155858 led to significant inhibition of SNU668 and MKN1 tumor growth compared with controls (Figure 3A and 3B). However, the AR-C155858 failed to exert any anti-tumor activity on SNU620 xenografts (Figure 3C). Immunohistochemistry (IHC) was performed using a monoclonal anti-PCNA antibody for validation of tumor growth inhibition with AR-C155858 in xenografts, as shown in Figure 3D. The brown staining in the nucleus, which is the PCNA signal, was decreased in SNU668 and MKN1 xenografts treated with AR-C155858. In contrast, AR-C155858 did not affect PCNA expression in SNU620 xenografts.

Although subcutaneous models are well established and widely used in GC experimental studies, these models may not completely represent the biologic characteristics of GC [24, 25]. Zhang et al. established a peritoneal dissemination xenograft mouse model for survival outcome assessment of experimental GC [26]. Given that peritoneal metastasis is the most frequent pattern of recurrence of GC [27], we next assessed tumor growth inhibition in peritoneal dissemination xenograft models. Starting the day after intraperitoneal injection of 1 × 10^7^ SNU668 cells in nude mice, the mice were treated 3 times per week with intraperitoneal injection of 3 mg/kg inhibitors. We found that intraperitoneal administration of AR-C155858 led to significant inhibition of tumor volume compared with controls (Figure 3E).

### Knockdown of MCTs synergistically increases cell death induced by chemo- and radiotherapy

**Figure 4:**
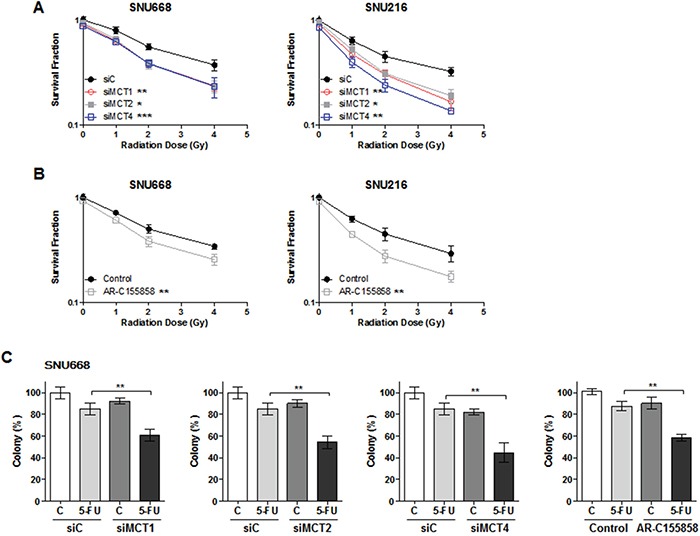
Synergism effect of MCT inhibition with anti-cancer therapy SNU668 and SNU216 cells were transfected with the indicated siRNAs or treated with AR-155858. Twenty-four hours later, the cells were irradiated with a ^137^Cs source **A.** and **B.** or 5-fluorouracil **C.** Surviving fractions following the given treatments were calculated based on the survival of nonirradiated cells. * *P* < 0.05, ** *P* < 0.01, *** *P* < 0.001.

We next investigated the synergy between MCT inhibition and radiotherapy or clinically relevant cytotoxic drugs (Figure 4). SNU668 and SNU216 cells were transfected with MCT siRNA or treated with AR-155858. Twenty-four hours later, the cells were irradiated with a ^137^Cs source or treated with 5-fluorouracil (5-FU).

We observed radiation dose-dependent inhibition of cell proliferation that was significantly more suppressed upon knockdown of MCT1, 2, or 4 compared with siC (Figure 4A). The same phenomenon was also observed in cell lines treated with AR-C155858 (Figure 4B). Down-regulation of MCTs significantly increased cell death induced by 5-FU regardless of type of MCT siRNA (Figure 4C). In addition, AR-C155858 enhanced the antitumor effect of 5-FU in GC cell lines (Figure 4C).

### MCT1, 2, and 4 are differentially expressed in normal tissue, tumor tissue, and malignant ascites

We next characterized the MCT expression levels in 45 primary GC tissues along with the corresponding normal gastric tissue using real-time PCR. The mRNA expressions were well correlated with protein expression levels using western blot analysis (Supplementary Figure S2). We also screened 48 patient-derived cells (PDCs) collected from malignant ascites for the expression of MCTs. The clinical and pathologic features for the patients are summarized in Table 1.

**Table 1:** Baseline characteristics

	Tumor tissue (n = 45)		PDC (n = 48)	
	No.	%	No.	%
Median age (range)	64.1 (32.3-81.9)		50.7 (26.8-78.3)	
Sex				
Male	37	82.2	25	52.1
Female	8	17.8	23	47.9
Stage†				
II	14	31.1	3	6.3
III	22	48.9	7	14.6
IV	9	20.0	38	79.2
Location				
Proximal	5	11.1	4	8.3
Body	26	57.8	22	45.8
Antrum	13	28.9	18	37.5
Diffuse	1	2.2	4	8.3
Histology‡				
Tubular adenocarcinoma	40	88.9	35	72.9
Well differentiated	0	0	2	4.2
Moderate differentiated	12	26.7	6	12.5
Poorly differentiated	28	62.2	27	56.3
Signet-ring cell carcinoma	5	11.1	13	27.1

PDC, patient-derived cell

† Initial stage of diagnosis with gastric cancer

‡ The 2010 WHO classification

**Figure 5:**
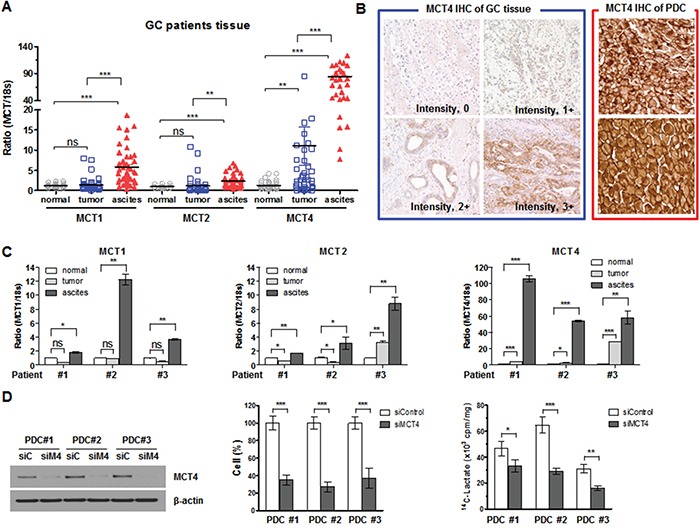
MCTs expression in GC tissues **A.** MCT1, MCT2, and MCT4 mRNA and 18S rRNA were detected using real-time PCR according to tissue (normal tissue, tumor tissue, and PDCs collected from malignant ascites). Data were normalized using 18S rRNA as an endogenous control. **B.** MCT4 immunostaining of gastric cancer tissues and PDCs. Intensity MCT4 immunostatining was measured as score 0 (negative), score 1 (weak), score 2 (moderate), and score 3 (strong) for positive cases of gastric cancer tissue (left panel). Right panel represents two cases of PDCs with highly strong MCT4 expression. Original magnification, x 400. **C.** Three sets of MCT expression in patient-matched tissue. **D.** MCT4 expression in PDCs collected from malignant ascites and the effects of its silencing on cell proliferation and lactate uptake. * *P* < 0.05, ** *P* < 0.01, *** *P* < 0.001.

Comparisons of MCT expression measured by RT-PCR among the normal gastric tissue, primary GC tissue, and PDCs are depicted in Figure 5A. There was no significant difference in MCT1 and MCT2 expression between primary GC tissue and matched normal gastric tissue samples (Figure 5A). However, MCT4 expression was significantly increased in primary GC tissue compared with normal gastric tissue. In PDCs, MCTs were strongly overexpressed compared with normal tissue or primary GC tissue regardless of isoform (Figure 5A). Among the MCT isoforms, MCT4 was especially highly up-regulated in PDCs. Figure 5B represents immunostaining of MCT4 in gastric cancer tissues and PDCs. We found that PDC samples showed extremely strong MCT4 expression (Figure 5B). Figure 5C shows patient-matched MCT expression levels in normal tissue, GC tissue, and malignant ascites. As can be seen, significant differences were observed for MCT4 expression (Figure 5C). We observed that siMCT4 were effective in down-regulating MCT4 expression in PDC cells (#1, #2, and #3) and the cell viability and uptake of lactate was significantly inhibited in cells treated with siMCT4 (Figure 5D).

## DISCUSSION

Most solid tumors are known to rely on glycolysis for energy production and this activity contributes to the acidic microenvironment of the tumor [28, 29]. The up-regulation of MCTs in cancers is an adaptive mechanism to allow continuous high glycolytic rates by regulation of pH, therefore having an important effect on cancer cell viability [29, 30]. A number of original studies that investigated the clinical and prognostic significances of MCTs have been conducted in various types of cancer [14, 31–35]. However, the expression of MCTs and its clinical value in GC are still poorly understood. Our results provided the first evidence that MCT4 might be a potential therapeutic target for GC with peritoneal carcinomatosis.

Expression patterns of MCTs vary with cell type. MCT1 is expressed in almost all tissues, whereas MCT4 is expressed mainly in glycolytic tissues (*e.g.* white muscle) [36] and MCT2 is absent in most human tissues [36]. With relevance to cancer, there is considerable variation in the MCT isoforms expressed in different tumors [31, 37, 38]. Compared with normal tissue, MCT1 and MCT4 are up-regulated in breast cancer, while MCT4 is down-regulated in lung cancer [31]. In the current study, MCT1 expression was noted in multiple GC cell lines, while little expression of MCT2 was observed. It was interesting to find that MCT4 expression was mainly observed in the GC cell lines that originated with metastasis and malignant ascites.

Based on the patterns of MCT expression in GC cell lines, we explored the function of MCTs *in vitro* and *in vivo*. Down-regulation of MCTs reduced cancer cell proliferation as well as lactate uptake in a subset of GC cell lines that overexpressed MCTs. In mouse xenograft models derived from a GC cell line, AR-C155858 consistently inhibited MCT1, 2, and 4 co-expressing tumor growths, but had no impact on growth of tumors that expressed only MCT4. We found that in SNU668 cells, which co-express MCT1, 2 and 4, the influx of lactate was significantly inhibited by the individual siMCTs or AR-C155858. On the other hand, Draoui et al. showed that cervix cancer SiHa cells, which express both MCT1 and MCT4, the AR-C155858 compound failed to block lactate influx [39]. These findings might suggest that there are variations of lactate uptake through MCTs among the cell lines. Further studies of the capacity to target lactate influx independently of the type of MCT expression are needed.

Remarkably, targeting MCTs enhanced the antitumor activities of other therapeutic modalities, such as radiotherapy and chemotherapy, when used in combination. Hypoxia contributes significantly to tumor progression and resistance to radiotherapy or chemotherapy [40, 41]. According to Sonveaux et al., the metabolic switch induced by tumor hypoxia accounts for reduced oxygen consumption by the surviving tumor cells, thereby resulting in tumor radiosensitization after MCT1 inhibition [42]. In a recent study by Chadwick et al. MCT4 expression was found to be a prognostic factor for radiotherapy outcome in head and neck squamous cell carcinoma [43]. Recently, Amorim et al. reported that 5-FU cytotoxicity was potentiated by lactate transport inhibition in colorectal cancer cell lines, either by activity inhibition or expression silencing of MCT [44]. Collectively, these results favor the hypothesis that MCTs might be considered a potential therapeutic target for treatment of GC.

High expression of MCT4 was found in ‘glycolytic’ tissues, including several hypoxic and rapidly growing tumors [29], such as gliomas [17]. We identified a significantly higher level of MCT4 expression by using real-time PCR in primary GC tissue, when compared to the corresponding normal gastric tissue. However, the same was not observed for MCT1 and MCT2. It is noteworthy that MCT4 displays extremely strong expression, especially in PDCs collected from malignant ascites. Considering that MCT expression is influenced by altered physiologic conditions [45], it might be that hypoxia-induced metabolic adaptations lead to up-regulation of MCT4 expression in GC with peritoneal seeding [9, 46].

To better understand the prognostic value of MCT4 in GC patients, we assessed MCT4 expression by IHC in a large GC cohort of the ARTIST trial, which has been published previously (Supplementary Table S1) [47]. Contrary to previous studies that the MCT4 expression significantly correlated poor prognosis [48–51], MCT4 expression did not correlate with prognosis in our study (Supplementary Table S2 and Supplementary Figure S3). Several reasons may explain the contradictory findings the MCT4 expression and clinical outcomes. One of the most possible reasons is the selection bias that only explored the primary GC tissues from the patients with curative resection. Analysis of the MCT4 expression in metastatic site or malignant ascites could have a more precise reflection of prognosis. It is also worth noting that these studies were based on the different methods of IHC (Supplementary Table S3) such as heterogeneous antibodies and the arbitrary scoring system. For accurate and reliable assay to assess MCT4 by IHC, commercially available reagents and validated methodologies are needed.

Peritoneal carcinomatosis in advanced GC remains an intractable clinical problem despite advances in the treatment of GC. Peritoneal dissemination is the most frequent pattern of gastric cancer recurrence (33-50%) [27, 52, 53]. In addition, over 10% of gastric cancer patients have peritoneal disseminations at the time of diagnosis [54]. The prognosis of GC patients with peritoneal dissemination is poor, with a median survival of less than 6 month due to the lack of effective treatments for these tumors [55]. Based on that the MCT4 isoform was very strongly expressed in malignant ascites, development of a highly potent MCT4 inhibitor might be effective for treatment of GC with peritoneal carcinomatosis. Our group has previously shown that PDCs are reflective of genomic alterations in patient tumors and clinical phenotypes in response to targeted agents [56]. To the best of our knowledge, this is the first study to systematically investigate the role of MCT4 in malignant ascites using PDCs.

Our results suggest that MCT4 plays a central role in tumor metabolism in GC with peritoneal carcinomatosis and targeting MCT4 in combination with chemotherapy could be a novel strategy in the treatment of GC.

## MATERIALS AND METHODS

### Study design

The objective of this study was to assess whether the MCTs could be a therapeutic target for treatment of advanced GC patients. First, we examined the expression levels of MCTs including MCT1, 2, and 4 in human GC cell lines by reverse transcription polymerase chain reaction (RT-PCR) and western blot. Second, to investigate the functional role of MCTs in GC, we explored the effect of knockdown of MCTs on cell proliferation and lactate uptake. Third, we examined whether inhibition of MCTs could translate in *in vivo* GC xenograft models and enhance the anti-tumor activity of radiotherapy or conventional chemotherapy. Fourth, for identifying the significant differences in MCT expression between normal tissue and tumor tissue, we compared the levels of MCT expression of normal gastric tissue, primary GC tissue, and PDCs derived from malignant ascites by using real-time PCR.

### Ethics statement

This study was approved by the Samsung Medical Center Institutional Review Board (Seoul, South Korea) in accordance with the ethical principles of the Declaration of Helsinki and local guidelines. Written informed consent was obtained from all participating patients.

### Cell culture

We screened 16 established GC cell lines including 6 primary cell lines (AGS, OCUM-2M, MKN45, SNU1, SNU484, and SNU719), 4 metastatic cell lines (MKN1, MKN28, MKN74, and SNU216), and 6 ascite cell lines (SNU5, SNU19, SNU601, SNU620, SNU638, and SNU668) in order to identify the cell lines that expressed high levels of MCT1, 2, and 4.

### RT-PCR and western blot analyses

Total cellular RNA was extracted using an RNeasy MiniKit (Qiagen) and treated with DNase I. One microgram of RNA was converted to cDNA using an Omniscript RT Kit (Qiagen). Real-time PCR was performed by using a Prism 7900HT Sequence Detection System (PE Applied Biosystems).

For western blot analyses, antibodies against MCT1 (Santa Cruz Biotechnology, H-1, 1:1000), MCT2 (Atlas Antibodies, HPA005911, 1:400), MCT4 (Santa Cruz Biotechnology, H-90, 1:500), and β-actin (Sigma, AC-15, 1:5,000) were used. The protocols used are described in the Supplementary Materials.

### RNA interference and transfection

Silencing of MCT expression was done with siRNA targeting MCT1, MCT2, and MCT4 (Dharmacon, Lafayette) using HiPerfect transfection reagents (Qiagen) according to the manufacturer's instructions.

### Cell growth assessment and colony formation assay

To assess cell numbers, cells (1 × 10^5^ cells per 6-well plate, Corning) were transfected with siRNAs and incubated for 3 days. Cell proliferation following each treatment was compared with untreated cells. For the clonogenic assay, cells were transfected with siRNAs for 24 hours, irradiated with a ^137^Cs source (2.01 Gy/min, IBL-437C, CIS-US Inc.), incubated for 14 days, and then colonies were counted. All these experiments are described in detail in the Supplementary Materials.

### [^14^C]-Lactate uptake assays

For lactate uptake measurements, siRNA-transfected cells were equilibrated in buffered solution adjusted to pH 6. [^14^C]-L-lactate (Amersham Biosciences) was expressed as counts per million per milligram of protein. The protocols used are described in the Supplementary Materials.

### Xenograft study

To determine whether an MCT inhibitor has antitumor effects *in vivo*, we implanted mice subcutaneously or intraperitoneally with GC cell lines and assigned the animals to two groups based on their treatment with PBS only or AR-C155858. All mice experiments were conducted in accordance with the Institute for Laboratory Animal Research Guide for the Care and Use of Laboratory Animals, and the protocols were approved by the appropriate Institutional Review Boards at Samsung Medical Center (Agreement- 20141211001). Xenograft models of GC are described in the Supplementary Materials.

### Human gastric cancer and PDCs

Forty-five matched pairs of primary GC and normal gastric tissue and PDCs (N = 48) were collected at the Samsung Medical Center. All GC tumors and control tissues were confirmed by the hospital's clinical pathology department. To establish PDCs from metastatic GC patients with malignant effusion, those who were enrolled in the SMC Oncology Biomarker study (NCT#01831609) were screened for the expression of MCTs by western blot. Details of cell lines and cell cultures are provided in the Supplementary Materials.

All patients provided written informed consent. This study was performed in accordance with the Declaration of Helsinki and was approved by the Institutional Review Board of Samsung Medical Center.

### MCT4 immunohistochemistry

We examined *MCT4* expression status in patients enrolled in the ARTIST phase III clinical trial [47]. All slides were reviewed by a pathologist (K.-M.K.), and the immunohistochemical stains (IHC) for MCT were performed according to the protocols. Levels of expression were scored semiquantitatively by assessing the average signal intensity (on a scale of 0 to 3) and the proportion of tumor cells showing a positive signal (on a scale of 0 to 3). Details of IHC are described in the Supplementary Materials.

### Statistical analysis

Preclinical data were evaluated by a two-tailed *t*-test or *one-way ANOVA* using GraphPad Prism version 4.01 (San Diego, CA, USA). For analysis of the MCT4 IHC cohort, SPSS statistical software version 20 (Chicago, IL, USA) was used. All comparisons were examined by the *χ^2^* test or Fisher exact test. The Kaplan-Meier test was used to evaluate the survival rate and survival curves were compared by the log-rank test. A Cox proportional hazard model was applied for multivariate survival analysis. All tests were two-tailed, and P values < 0.05 were considered significant.

## SUPPLEMENTARY DATA




