# Supplementary material for: MCT4 as a potential therapeutic target for metastatic gastric cancer with peritoneal carcinomatosis
Source: Oncotarget. 2016 May 20;7(28):43492–503. doi: 10.18632/oncotarget.9523 (PMC5190039; doi:10.18632/oncotarget.9523)
Supplement: Supplementary file 1 [file oncotarget-07-43492-s001.pdf]

SUPPLEMENTARY DATA

Cell culture and establishing PDCs

GC cell lines including (6 primary cell lines: AGS, OCUM-2M, MKN45, SNU1, SNU484, SNU719; 4 metastatic cell lines: MKN1, MKN28, MKN74, SNU216; ascites cell lines: SNU5, SNU19, SNU601, SNU620, SNU638, SNU668) were used. GC cell lines were purchased from the American Type Culture Collection (ATCC) and the Korean Cell Line Bank (KCLB).

To establish PDCs from metastatic GC patients with malignant effusion, those who were enrolled onto the SMC Oncology Biomarker study (NCT#01831609) were screened for the MCTs expression by Western blot. All patients provided informed consent form according to the SMC Institutional Review Board. Briefly, collected effusions (1–5 L) were divided into 50 mL tubes, centrifuged at 1500 rpm for 10 min, and washed twice with PBS.

All cells and PDCs were grown in RPMI-1640 medium supplemented with 10% FBS, an antibiotic, and an antimycotic. Cells were cultured at 37°C in a humidified 5% CO_2 environment.

RT-PCR

Total cellular RNA was extracted using RNeasy MiniKit (Qiagen) and treated with DNase I (Qiagen). Onemicrogram ofRNAwas converted to cDNA using Omniscript RT Kit (Qiagen). The primer sequences designed from the coding region of human MCT cDNA are as follows: MCT1 cDNA forward, 5'-GGAAGGTGGACCAGAATGAA-3' and reverse, 5'-CAATTTAGCAAGGCCCAAAA-3'; MCT2 cDNA forward, 5'-AGGATTAATTGCAAACTCCA -3', and reverse, 5'- CCGAATGTTTAGATTTGCTC-3'; MCT4 cDNA forward, 5'-TGGGATGGGACTGACTTTTC-3' and reverse, 5'-AGCAGTTTGTCTGCACATGG-3'. The PCR conditions were as follows: 30 cycles of 95°C for 30 seconds, 55°C for 30 seconds, and 72°C for 30 seconds.

Real-time PCR was performed by using a Priam 7900HT Sequence Detection System (PE Applied Biosystems). MCT1, MCT2, MCT4 mRNA and 18S rRNA were detected, using TaqMan Gene Expression Master Mix Reagent and TaqMan probe (Applied Biosystems). Data were normalized using 18S rRNA as an endogenous control and calculated using the comparative Ct method (2-delta delta Ct).

Western blot analyses

Total cell extracts were obtained using lysis buffer (20 mM HEPES [pH 7.4], 1% Triton X-100, 1 mM EDTA, 1 mM $MgCl_2$, 150 mM NaCl, 10% glycerol, and protease inhibitor cocktail [Invitrogen]), and protein concentration was determined using the micro-BCA protein reagent (Pierce Biotechnology). Equal amounts of proteins (30 µg per well) from the clarified lysates were separated by sodium dodecyl sulfate–polyacrylamide gel electrophoresis and transferred onto nitrocellulose membranes having a 0.45-µm pore size (Whatman). The membranes were sequentially incubated in 5% dry milk and antibodies against MCT1 (Santa Cruz Biotechnology, H-1, 1:1000), MCT2 (Atlas antibodies, HPA005911, 1:400), MCT4 (Santa Cruz Biotechnology, H-90, 1:500), and β-actin (Sigma, AC-15, 1:5,000). The ECL system was used for protein detection (Invitrogen).

RNA interference and transfection

The 21-nucleotide-long siRNAs targeting MCT1, MCT2, MCT4, and negative control siRNA (siC) were purchased from Dharmacon. Cells (3×10^5 cells per 60 mm dishes) were transfected with 20 nM siRNAs using HiPerfect transfection reagents (Qiagen) according to the manufacturer's instructions and were used for western blot analysis 48 hours after transfection. Sequences of the siRNAs used were MCT1-targeted siRNA (siMCT1) (5'- CCAAGGCAGGGAAAGAUAAGUCUAA-3'), MCT2-targeted siRNA (siMCT2) (5'-GGAUUUAACUGGAGAAUAU-3'), MCT4-targeted siRNA (siMCT4) (5'-CGACCCACGUCUACA UGUACGUGUUUU-3'), and control nontargeting siRNA (5'-UAGCGACUAAACACAUCAA-3').

Cell growth assessment and colony formation assay

To assess cell numbers, cells (1×10^5 cells per 6-well plate, Corning) were transfected with siRNAs and incubated for 3 days. Adhered cells were trypsinized, stained with 0.2% trypan blue (Sigma) and counted using a hemocytometer. Cell proliferation each treatment was compared with untreated cells.

For the clonogenic assay, cells were transfected with siRNAs for 24 hours, irradiated with a ^{137}Cs source (2.01 Gy/min, IBL-437C, CIS-US Inc.), trypsinized

and counted. 400 cells were replated in 6-well plate. After incubation at 37°C for 10 ~ 14 days, colonies were stained with 0.005% (w/v) crystal violet (Sigma). Colonies (> 50 cells) were counted and surviving fractions following given treatments were calculated based on the survival of nonirradiated cells.

[^{14}C]-Lactate uptake assays

siRNA transfected or drug treated cells were equilibrated for 5 min in 20 mM Mes-buffered glucose-free and HCO_3-free saline solution containing 120 mM NaCl, 5 mM KCl, 2 mM $CaCl_2$, 1 mM $MgCl_2$, adjusted to pH 6. The solution was then replaced by the same solution containing 0.2 µCi/mL of [^{14}C]-L-lactate (Amersham Biosciences) and incubated for 10 min at RT. Uptake was stopped by washing four times with ice-cold PBS. Cells were lysed with 250 µL of 0.1 M NaOH. The radioactivity was determined by mixing 4 mL of scintillation liquid with 200 µL of cell lysate and counted with a liquid scintillation counter (PerkinElmer). The protein concentration was determined to normalize radioactivity to the protein content of the cells in each dish. [^{14}C]-L-lactate uptake was expressed as counts per million per milligram of protein.

Xenograft study

Male BALB/c nude mice, 4 ~ 6 weeks old, were obtained from Orient Bio Inc. Mice were implanted subcutaneously with SNU668 (1×10^7), MKN1 (1×10^7), or SNU620 (1×10^7) cells in 100 µl volume. The mice were randomized and the treatment started when the tumor size reached 30 mm^3 at 7 days after inoculation. Mice were assigned into two groups: PBS only or AR-C155858 (Chemscene). AR-C155858 (1 mg/kg, intraperitoneal injection) were given 3 times per week. The growth of tumors was measured using a digital caliper (Proinsa) every 2 ~ 3 days and average tumor volumes were calculated using the following formula: $V = (L \times W^2) / 2$, where V = volume (in cubic millimeters), L = length (in millimeters), and W = width (in millimeters). The mice were sacrificed, and the tumors (three tumors per treatment group) were resected and frozen in liquid nitrogen until later use for western blot analyses. All mice experiments were conducted in accordance with the Institute for Laboratory Animal Research Guide for the Care and Use of Laboratory Animals, and the protocols were approved by the appropriate Institutional Review Boards at Samsung Medical Center (Agreement- 20141211001).

For the peritoneal dissemination model, 1×10^7 SNU668 cells were inoculated intraperitoneally into male BALB/c nude mice. On the following day, the mice (n=5 mice per treatment group) were assigned into two groups: PBS only or AR-C155858. The mice were treated 3 times per week with intraperitoneal injection of 3 mg/kg inhibitors in PBS. After 3 weeks, animals were sacrificed, and tumor nodules were counted.

MCT4 immunohistochemistry

We examined *MCT4 expression* status in patients enrolled in the ARTIST phase III clinical trial.15 In brief, patients were stage IB to IV (M0) GC who had undergone curative D2 surgery between November 2004 and April 2008. Primary tumor tissues from 415 surgical specimens were available for tissue microarray.

Immunohistochemistry was performed according to avidin–biotin–peroxidase complex principle (R.T.U. Vectastain Elite ABC Kit (Universal), Vector Laboratories), with the primary antibodies for MCT4, used in Western blotting, diluted 1:200. Briefly, deparaffinized and rehydrated sections were incubated in 0.3% hydrogen peroxide for 30 min, to inactivate endogenous peroxidases, and washed in PBS. Antigen retrieval was performed by immersing slide-mounted sections in 0.01 M citrate buffered solution (pH 6.0) for 20 min in 97°C water bath and microwaving (600 W) for 15 min then washed in PBS. Tissue sections were then incubated with a protein blocking solution for 5 min and incubated with the primary antibody overnight at 4°C. Sections were then sequentially washed in PBS and incubated with biotinylated secondary antibody for 30 min, R.T.U. Vectastain Elite ABC reagent for 45 min at 37°C and developed with 3,3′-diamino-benzidine (DAB+ Substrate System, DakoCytomation) for 10 min. Negative controls were performed by using the adequate serum controls for the primary antibodies used (N1699 and X0907, DakoCytomation).

Sections were scored semi-quantitatively for immunoreactions extension as follows: score 0, 0%; score 1, <5%; score 2, 5–50%; score 3, >50%. Also, intensity of staining was scored semi-qualitatively as 0: negative; 1: weak; 2: intermediate; and 3: strong. Immunoreaction final score was defined as the sum of both parameters (extension and intensity), and grouped as negative (0), weak (1-2), moderate (3), and strong (4–6). For statistical purposes, grouped as negative (score 0-2) and positive (score 3-6). Figure 3 shows representative IHC for MCT4.

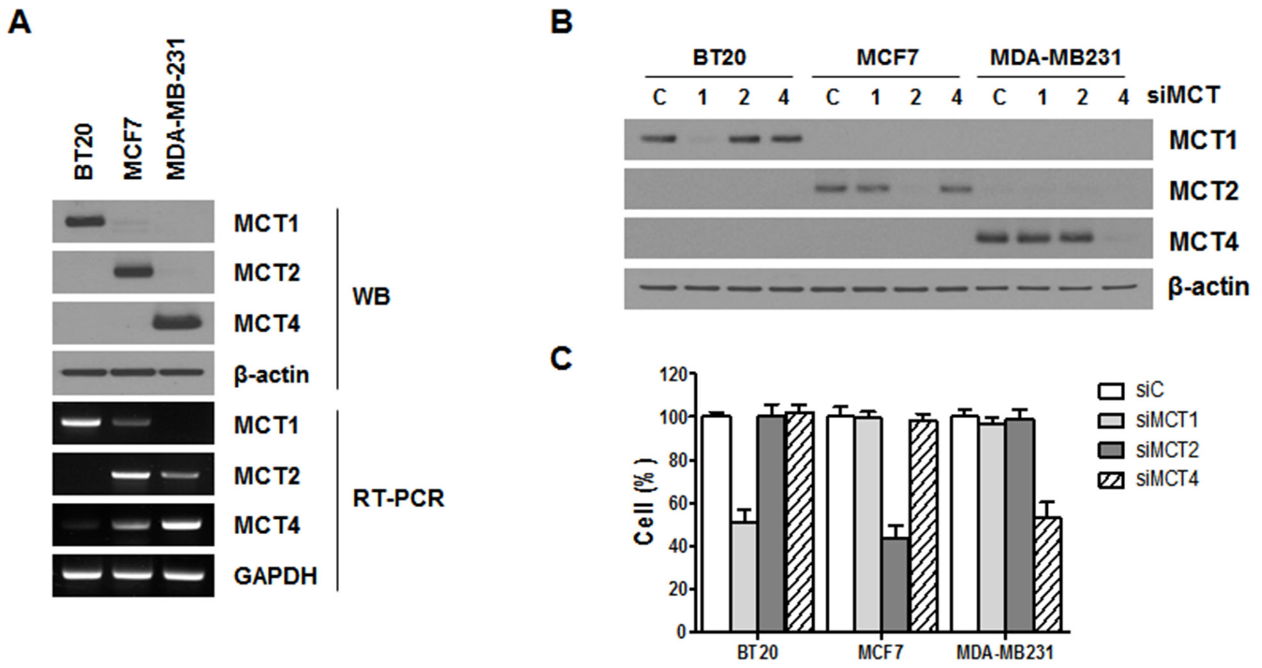

Supplementary Figure S1: MCT1, 2, and 4 expression in breast cancer cell lines and the effects of its silencing on cell proliferation. **A.** RT-PCR and western blot analysis was used to assess MCTs protein expression in 3 breast cancer cell lines (BT20, MCF, and MDA-MB231). **B.** Expression of MCTs in breast cancer cell lines after transfection of siRNAs was analyzed with western blot analysis using the antibodies shown on the right. **C.** Cell proliferation was measured 72 hours after transfection with MCTs siRNA (siMCT1, siMCT2, or siMCT4) and a negative control sequence (siC). The percentage of viable cells is shown relative to that of the untreated control.

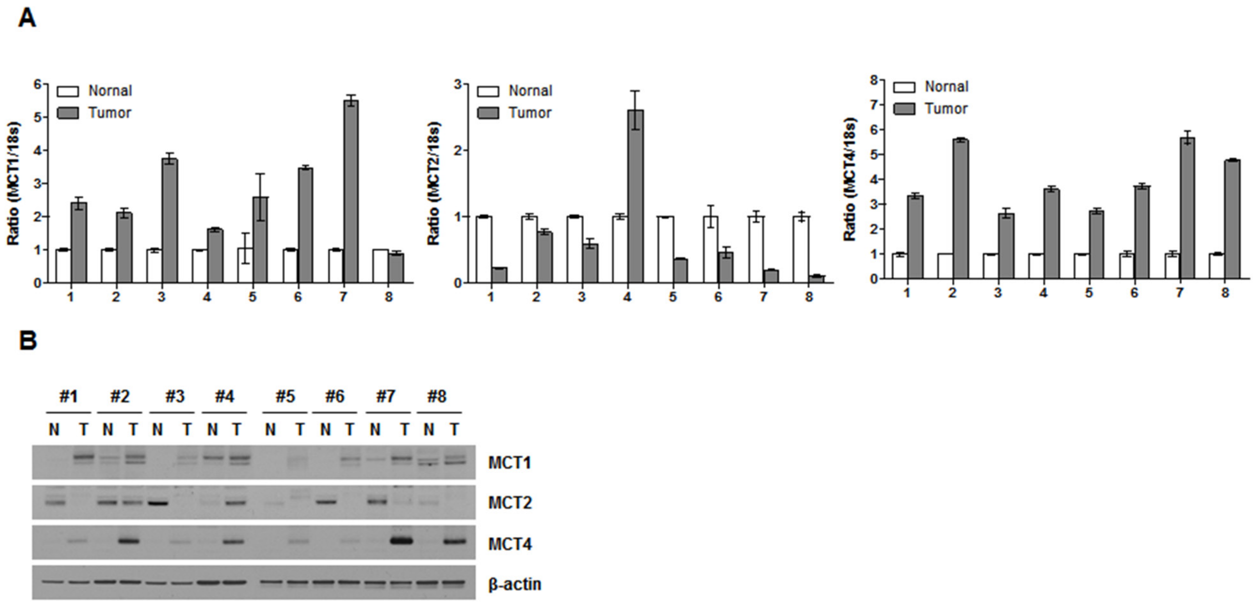

Supplementary Figure S2: Comparisons of MCTs expression between the normal gastric tissue and primary GC tissue using RT-PCR A. and western blot analysis **B.** in 8 GC patients. mRNA levels were normalized to 18s rRNA and protein levels to β-actin.

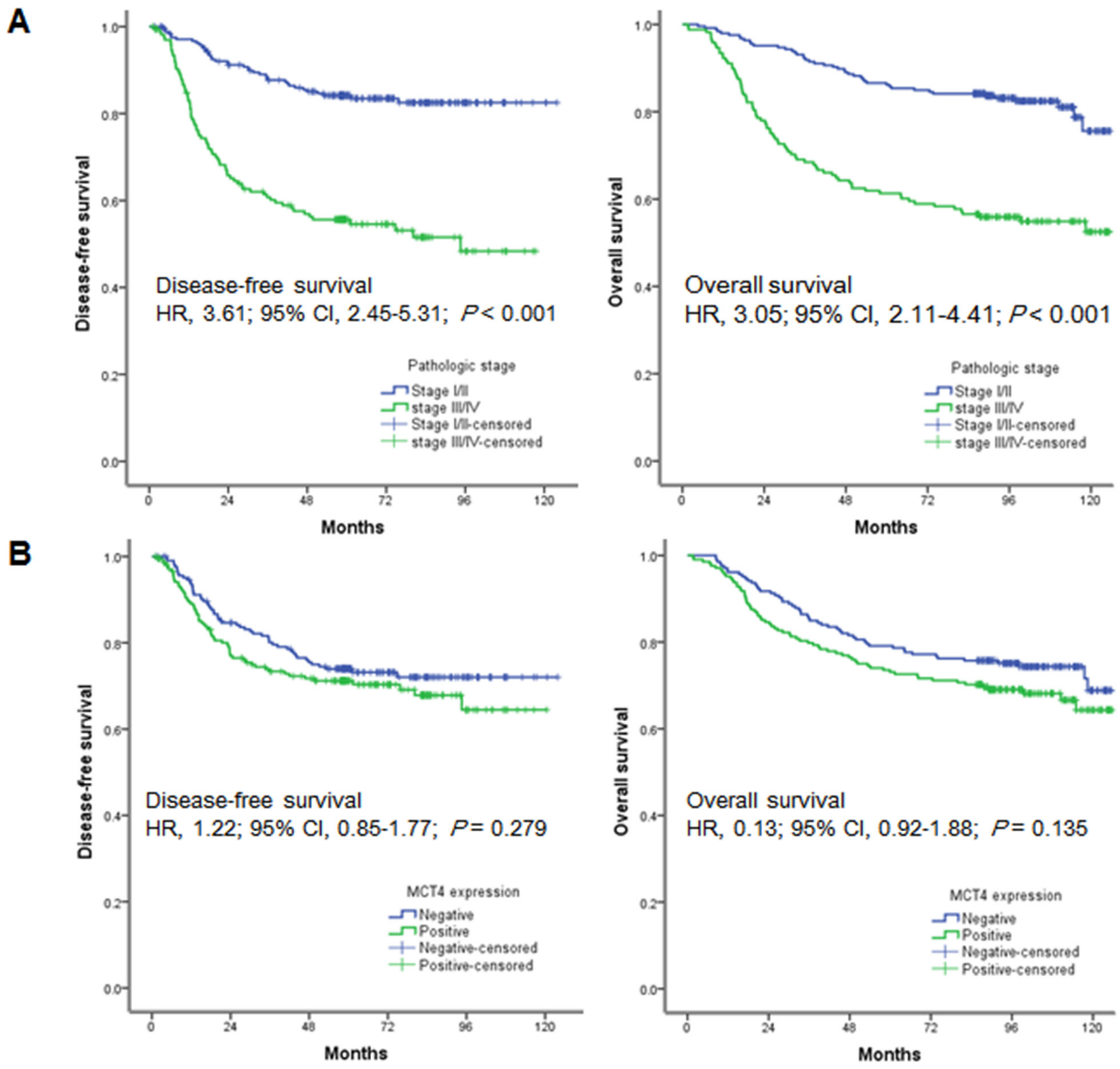

Supplementary Figure S3: Kaplan-Meier curves for disease-free survival and overall survival according to the stage A. and the MCT4 expression **B.**

Supplementary Table S1: Baseline characteristics

		Total (n = 415)		MCT4 Negative (n = 207)		MCT4 Positive (n = 208)		
		No.	%	No.	%	No.	%	*P*
Age	< 56	202	48.7	118	57.0	84	40.4	0.001
	≥ 56	213	51.3	89	43.0	124	59.6	
Gender	Male	270	65.1	127	61.4	143	68.8	0.114
	Female	145	34.9	80	38.6	65	31.3	
Location	Proximal	20	4.8	7	3.4	13	6.3	0.400
	Body	200	48.2	110	53.1	90	43.3	
	Antrum	157	37.8	73	35.3	84	40.4	
	Diffuse	38	9.2	17	8.2	21	10.1	
Lauren	Intestinal	150	36.6	53	26.0	97	47.1	0.000
	Diffuse/ mixed	260	63.4	151	74.0	109	52.9	
T stage	T1-2	306	73.7	155	74.9	151	72.6	0.597
	T3-4	109	26.3	52	25.1	57	27.4	
N stage	N1	289	69.6	147	71.0	142	68.3	0.543
	N2-3	126	30.4	60	29.0	66	31.7	
Stage	I-II	247	59.5	127	61.4	120	57.7	0.448
	III-IV	168	40.5	80	38.6	88	42.3	
LV invasion	Absent	142	34.6	82	40.2	60	29.1	0.019
	Present	258	65.4	122	59.8	146	70.9	
PN invasion	Absent	212	52.6	109	54.8	103	50.5	0.389
	Present	191	47.4	90	45.2	101	49.5	

Supplementary Table S2: Univariate and multivariate analysis of disease-free survival

	Disease-free survival			Overall survival		
	**HR**	**95% CI**	***P* value**	**HR**	**95% CI**	***P* value**
Age (≥ 56 years)	1.13	0.78-1.62	0.528	1.09	0.76-1.55	0.647
Sex (Female)	1.23	0.85-1.79	0.272	1.25	0.87-1.80	0.230
Lauren type (diffuse)	1.31	0.88-1.96	0.184	1.37	0.93-2.02	0.113
Histology (non tubular)	1.27	0.87-1.83	0.214	1.44	1.01-2.06	0.047
pStage (Stage III-IV, M0)	3.61	2.45-5.31	0.000*	3.05	2.11-4.41	0.000*
MCT4 (positive)	1.22	0.85-1.77	0.279	1.31	0.92-1.88	0.135

Supplementary Table S3: Immunostaing from recent studies of MCT4

See Supplementary File 1
