# Supplementary material for: MCT4 as a potential therapeutic target for metastatic gastric cancer with peritoneal carcinomatosis
Source: Oncotarget. 2016 May 20;7(28):43492–503. doi: 10.18632/oncotarget.9523 (PMC5190039; doi:10.18632/oncotarget.9523)
Supplement: Supplementary file 2 [file oncotarget-07-43492-s002.docx]

**Supplementary Table 3. Immunostaing from recent studies of MCT4**

|  | Country | Tumor | N | Disease status | Antibody | Intensity | Proportion | Cut-off, sum | Results |
| --- | --- | --- | --- | --- | --- | --- | --- | --- | --- |
| Pertega-Gomes et al.  [34] | Portugal | Prostate  cancer | 171 | Curative resection | AB3316P | 0, negative  1, weak  2, moderate  3, strong | 0, 0%  1, < 5%  2, 5-50%  3, > 50% | Negative, 0-3  intermediate, 4  high, 5-6 | Intermediate, 21%  High, 23% |
| Gotanda  et al.  [49] | Japan | Colorectal  cancer | 210 | Curative resection | Sc-50329 | 0, negative  1, weak  2, moderate  3, strong | 0, 0%  1, ≤ 5%  2, 6-49%  3, ≥ 50% | Positive, ≥ 4 | Positive, 49% |
| Gao et al.  [50] | China | Hepatocellular  carcinoma | 318 | Curative resection | Hpa021451 | 0, negative  1, weak  2, moderate  3, strong | 0, 0%  1, 1-25%  2, 26-50%  3, 51-75%  4, ≥ 76% | proportion  Low, 0-1  Medium, 2  High, 3-4 | Medium, 31%  High, 26% |
| Zhu et al.  [51] | China | Oral squamous  cell carcinoma | 99 | Curative resection | Sc-376140 | 0, negative  1, weak  2, moderate  3, strong | 0, < 5%  1, 5-10%  2, 10-50%  3, 50-75%  4, > 75% | High, ≥ 6 | High, 44% |
| Zhao et al.  [21] | China | Gastric cancer | 113 | Curative resection | sc-50329 | 0, negative  1, weak  2, moderate  3, strong | 0, 0%  1, < 5%  2, 5-50%  3, > 50% | High, ≥ 4 | High, 37% |
| Pinheiro  et al.  [35] | Portugal | Gastric cancer | 190 | NS | AB3316P | 0, negative  1, weak  2, moderate  3, strong | 0, 0%  1, < 5%  2, 5-50%  3, > 50% | Negative, 0-2  Positive, ≥ 3 | Positive, 18% |
| Lee et al. | Korea | Gastric cancer | 415 | Curative resection | sc-50329 | 0, negative  1, weak  2, moderate  3, strong | 0, 0%  1, < 5%  2, 5-50%  3, > 50% | Negative, 0-2  Positive, ≥ 3 | Positive, 50% |
